# Antidiabetics, Anthelmintics, Statins, and Beta-Blockers as Co-Adjuvant Drugs in Cancer Therapy

**DOI:** 10.3390/medicina58091239

**Published:** 2022-09-07

**Authors:** Laurentia Gales, Leyla Forsea, Diana Mitrea, Irina Stefanica, Irina Stanculescu, Radu Mitrica, Mihai Georgescu, Oana Trifanescu, Rodica Anghel, Luiza Serbanescu

**Affiliations:** 1 Department of Oncology, “Carol Davila” University of Medicine & Pharmacy, 022328 Bucharest, Romania; 2 Department of Oncology, “Prof. Dr. Alexandru Trestioreanu” Institute of Oncology, 022328 Bucharest, Romania; 3 Department of Radiotherapy, “Prof. Dr. Alexandru Trestioreanu” Institute of Oncology, 022328 Bucharest, Romania

**Keywords:** cancer therapy, metformin, mebendazole, atorvastatin, propranolol, beta-adrenergic receptors, drug repurposing

## Abstract

Over the last years, repurposed agents have provided growing evidence of fast implementation in oncology treatment such as certain antimalarial, anthelmintic, antibiotics, anti-inflammatory, antihypertensive, antihyperlipidemic, antidiabetic agents. In this study, the four agents of choice were present in our patients’ daily treatment for nonmalignant-associated pathology and have known, light toxicity profiles. It is quite common for a given patient’s daily administration schedule to include two or three of these drugs for the duration of their treatment. We chose to review the latest literature concerning metformin, employed as a first-line treatment for type 2 diabetes; mebendazole, as an anthelmintic; atorvastatin, as a cholesterol-lowering drug; propranolol, used in cardiovascular diseases as a nonspecific inhibitor of beta-1 and beta-2 adrenergic receptors. At the same time, certain key action mechanisms make them feasible antitumor agents such as for mitochondrial ETC inhibition, activation of the enzyme adenosine monophosphate-activated protein kinase, amelioration of endogenous hyperinsulinemia, inhibition of selective tyrosine kinases (i.e., VEGFR2, TNIK, and BRAF), and mevalonate pathway inhibition. Despite the abundance of results from in vitro and in vivo studies, the only solid data from randomized clinical trials confirm metformin-related oncological benefits for only a small subset of nondiabetic patients with HER2-positive breast cancer and early-stage colorectal cancer. At the same time, clinical studies confirm metformin-related detrimental/lack of an effect for lung, breast, prostate cancer, and glioblastoma. For atorvastatin we see a clinical oncological benefit in patients and head and neck cancer, with a trend towards radioprotection of critical structures, thus supporting the role of atorvastatin as a promising agent for concomitant association with radiotherapy. Propranolol-related increased outcomes were seen in clinical studies in patients with melanoma, breast cancer, and sarcoma.

## 1. Introduction

Prognoses are still dismal for advanced-stage cancer patients, despite the regular introduction of new molecules after lengthy clinical trials. Each case will go through a complex combination of surgery, radiotherapy, and chemotherapy following the decision of a multidisciplinary tumor board. Over the years, in oncology research, there has been growing evidence on the use of repurposed agents such as antimalarial, anthelmintic, antibiotics, anti-inflammatory, antihypertensive, antihyperlipidemic, and antidiabetic agents [1]. They have well-known properties and toxicity profiles, making them feasible for rapid implementation in new medical applications. In this review, we aimed to provide a scientific rationale for the use of metformin, mebendazole, atorvastatin, and propranolol as potential valuable co-adjuvants in cancer treatment. Frequently, for nonmalignant-associated pathologies, a combination of two or even three of the abovementioned drugs are present in the daily administration schedule of a given patient, throughout months or years of cancer therapy. Our patients’ desperate search for alternative cancer therapy often translates into the empiric use of drugs that they have within reach, with minimal scientific background. This review highlights the latest observational and preclinical data in the search for antitumor effects and the few clinical attempts to augment the oncological treatment with one of these four drugs of interest.

Metformin, a biguanide antihyperglycemic agent, is the most widely used first-line treatment for type 2 diabetes [2]. Thus, for many years it has been administered in diabetic patients bearing all other associated comorbidities including cancer. The common dosage is 500–2000 mg/day for its antidiabetic indication. The role of metformin has grown in recent times because of the vast number of epidemiologic studies that have been conducted on its potentially positive effects in decreasing cancer incidence and mortality. Significant benefits have been found, especially in diabetic patients with early-stage colorectal cancer and early-stage prostate cancer, often relating to an increased response to radiotherapy [3,4].

Mebendazole is an FDA-approved benzimidazole with a good safety profile, which has been widely used to treat parasitic diseases, mainly due to the fact of its tubulin binding capacity and prevention of microtubule polymerization. As an anthelmintic, common presentation of mebendazole is in 100 mg tablets, with indications for 100 mg as a single dose to 100–200 mg/day. Its mechanism of action has been linked to in vitro antiproliferative activity against cancer cells [5], but preclinical anticancer research also identified its ability to bind and inhibit signaling of selective tyrosine kinases including VEGFR2, TNIK, and BRAF [6]. It crosses the blood–brain barrier and evidence supports the use of mebendazole for both therapeutic and preventive purposes in the treatment of brain and colon cancers. [7,8]. Mebendazole is now being tested in clinical studies for glioblastoma, pediatric brain tumors, colon cancer, pancreatic cancer, thyroid cancer, prostatic cancer, and breast cancer [9,10,11].

Atorvastatin is a member of the statin family that inhibits HMG-CoA reductase activity, thus reducing cholesterol levels through inhibition of the mevalonate pathway at daily doses between 40 and 80 mg. It exhibits anticancer activity in several human cancer cells, mainly due to the fact of its autophagy-inducing effect [12,13,14,15]. On the other hand, several studies demonstrated the radioprotective effect of atorvastatin on normal tissues [16,17,18].

Various antihypertensive drugs influence the evolution of cancer such as calcium channel blockers, which stimulate apoptosis in the myeloma cell line and an autophagy-like process in prostate and colon cancer; angiotensin receptor blockers and their role in the upregulation of antitumoral T-cell lines; angiotensin-converting enzyme inhibitors revealing evidence of the downregulation of VEGF transcription. Beta-adrenergic receptors have been associated with signals involved in tumor evolution [19]. As a nonspecific inhibitor of beta-1 and beta-2 adrenergic receptors, propranolol was one of the first beta-blockers used in cardiology for arterial hypertension control, dysrhythmias, migraines, anxiety-associated symptoms, with daily doses of approximately 80 mg/day. It is also used in vascular tumors, such as infantile hemangiomas, but growing evidence suggests an emerging role in the treatment of vascular sarcomas [20]. Table 1 provides an overview of the main pre-clinical and clinical results after the use of the four drugs.

## 2. Metformin

Even though metformin has a long history of being used as an antidiabetic drug (derived from the plant *Galega officinali*), its mechanism of action was only recently discovered [21]: a direct one via the inhibition of mitochondrial ETC/OxPhos and the consequent activation of AMPK [22]; an indirect mechanism by lowering systemic insulin levels (via inhibition of hepatic gluconeogenesis) [21,23].

The rationale behind metformin’s proposed antitumor effect:

- Insulin stimulates cellular proliferation, and multiple signaling pathways are activated after insulin receptors or IGF-I receptors interact with their ligands; lowering insulin levels in the host blood removes one of the tumor-stimulating pathways [3,24];
- Metformin stimulates cancer cell apoptosis through activation of the AMPK/LKB1/TORC1 signaling pathway and inhibition of mTOR activity;
- Metformin counteracts metabolic disturbances known to influence tumor behavior such as obesity and insulin resistance. Other important factors are metformin’s anti-inflammatory effects;
- Metformin increases tumor radiosensitivity through mitochondrial ETC inhibition of oxygen consumption, thus improving tumor oxygenation;
- Metformin increases tumor radiosensitivity through the downregulation of the EGFR/PI3K/Akt signaling pathway, which further leads to mTOR inhibition, cell cycle arrest, and repression of colony formation ability [4,25,26].

Observational studies revealed that diabetic patients treated with metformin had a lower incidence of any kind of cancer in a dose-dependent manner compared to patients on other treatments. Coyle et al. conducted an analysis of 27 observational studies (24,178 participants), and the results regarding the tumor-related efficiency of metformin were mixed. A more beneficial approach for diabetic patients with prostate cancer may come from radical radiotherapy combined with metformin, whereas for early-stage colorectal cancer, metformin provided a clear benefit [3].

As shown in a complex study conducted by Zanella et al., first an in vitro analysis of various carcinoma cell lines revealed a significant decrease in oxygen consumption after metformin treatment, with the prostate cell line, LNCaP, being the most metformin-sensitive. This result represents an alternative method to improving tumor oxygenation for higher radiosensitivity. Second, mice bearing xenografts that were administered metformin 30 min before irradiation (100 mg/kg metformin equivalent to approximately 25% of the daily oral dose taken by patients with diabetes) revealed an improved oxygenation of the tumor and improved in vivo response to radiotherapy. In the same study, a retrospective clinical analysis of 504 prostate cancer patients identified 114 patients taking metformin (dosage not available) at the time of radiotherapy. For these patients, metformin use was a significant and independent factor reducing early biochemical relapse after radiotherapy [4].

Regarding advanced prostate cancer, neo-adjuvant radiotherapy is usually successful, but radioresistance is a key issue in many patients. In Zhang’s study, the investigators researched the effect of metformin on tumor cell radiosensitivity in prostate cancer. Using clonogenic survival assays, the team discovered that metformin enhanced radiosensitivity in prostate cancer cells. Moreover, there was an increase in radiation-induced tumor growth delay in mice with irradiated subcutaneous tumors. Additionally, mice with subcutaneous tumor implants had a longer life span after the combination of metformin and radiation. The reduced phosphorylation of DNA-PKcs caused by the downregulation of the EGFR/PI3K/Akt signaling pathway was essential for metformin to induce radiosensitivity in prostate cancer cells [27].

Iliopoulos et al. found metformin in combination with doxorubicin worked better than either drug alone at blocking prostate cancer growth as well as inhibiting relapse in mouse xenograft models. Because of this, doxorubicin doses can be lowered with milder toxicity from this medication with better tolerability and results. Similar results concerning the above combination were also seen in breast cancer cells with metformin plus a variety of chemotherapeutic agents (i.e., paclitaxel, carboplatin, and doxorubicin) [28]. This type of regimen has been seen to prevent relapse in xenografts generated with multiple types of breast cell lines. CSCs are a subset of tumor cells that are resistant to many anticancer treatments and are able to self-renew and regenerate various cell types that make up a tumor [29]. Prostate cancer stem cells are resistant to many of the usual cancer therapies, and it is believed that they contribute to local invasion and bone metastasis [30]. Underlying the fact that metformin selectively kills CSCs, researchers genetically manipulated human breast epithelial cells to enrich for stem cells and tested these together with three distinct breast tumor cell lines [31]. They discovered that metformin is selectively toxic to CSCs. To test metformin’s action in vivo, mice were implanted with transformed mammary epithelial cells and treated with three cycles of metformin and with doxorubicin. In this combination, metformin erased the tumors and prevented recurrence. No CSCs were recovered after the treatment and this response was sustained for approximately two months. The results were poorer when using only one agent, as metformin had no antitumor effect and doxorubicin revealed only an initial tumor shrinkage followed by relapse [32].

Type 2 diabetes is associated with increased incidence of colorectal cancer because of the hyperglycemic characteristic of the cancer cells, which require high glucose usage to compensate for the high metabolic activity [33,34]. A recent study concerning diabetics with stage of I to III colorectal cancer, who take metformin versus non-metformin diabetics, revealed a lower mortality in the first category [35]. The overall survival among patients with colorectal cancer and type 2 diabetes, who took metformin (dose not available), was better than patients who did not take this antidiabetic [36], but other authors recommend caution in interpreting these results—studies developed within an exclusive diabetic population may be biased due to the differences in the severity of diabetes between better prognosis populations with a first-line metformin approach and patients receiving other non-metformin antidiabetics [37]. However, for patients with thyroid carcinoma, it is not just the severity of associated morbidities that influences prognostics but also the direct relationship between the high glucose environment and the metformin’s antitumor effect. Metformin in a high-glucose environment inhibited cell proliferation and induced cell death in a low-glucose environment; therefore, the authors simply increased the cell sensitivity to metformin by adding a glycolysis inhibitor [38].

Many in vivo studies have been conducted to investigate the benefits of metformin, and the results are promising in regard to the growth of intestinal polyps. In mutated mice, high-dose metformin treatment for 10 weeks (250 mg/kg/day) inhibited the size of polyps but not the total number of intestinal polyps [34]. Given that many of the reports of metformin being used in the treatment of colorectal cancer are mostly centered among diabetic patients, we cannot safely say that it is, indeed, beneficial for nondiabetic patients too. The follow-up periods in the studies regarding colorectal cancer were very short; therefore, clinical studies that focus on late stages of colorectal cancer with patients using metformin for a longer period of time would offer a better view of the protective effect of metformin [34].

High-grade gliomas, especially glioblastomas, are among the most aggressive primary tumors that mandate the urgent development of new therapies to increase the efficacy of radio/chemotherapy. In vitro studies using metformin reported decreased proliferation, cell cycle arrest, autophagy, apoptosis, and cell death with a concomitant activation of AMPK, Redd1, and inhibition of the mTOR pathway [39].

Lately, clinical trials on the use of metformin in oncology have begun to report results. One of the very few randomized clinical trials on this topic, the OCOG-ALMERA, enrolled patients with locally advanced lung cancer for metformin at 2000 mg/day during chemoradiotherapy and afterwards for up to 12 months. With only 54 randomized patients, the study stopped early due to poor accrual. The results revealed worse local control and increased side effects associated with metformin use [40].

Another lung cancer trial, an open-label phase 2 study, NRG-LU001, enrolled 167 nondiabetic patients with unresectable stage 3 non-small cell lung cancer to receive carboplatin and paclitaxel-based chemoradiation either alone or with metformin (2000 mg/day after a gradual increase over 2 weeks, from 1000 mg/day in the first week). Interestingly, the study team reported an unexpected better 1-year PFS in the control arm than the one achieved in the PACIFIC reference study with durvalumab after chemoradiotherapy. However, the metformin arm, although well tolerated, did not bring any significant differences in the rates of survival, locoregional recurrence, or distant metastasis [41].

The largest clinical trial concerning metformin use, MA. 32, was a phase 3, double-blind trial that enrolled 3649 nondiabetic patients, with high-risk nonmetastatic, operable, breast cancer, randomized to receive standard therapy in association either with metformin at 850 mg twice a day or a placebo. Again, for most of the patient subcategories, there were no significant differences in disease-free survival and mortality. Grade 3 nonhematological toxic events, such as hypertension, irregular menses, and diarrhea, occurred more frequently in patients taking metformin than in patients taking the placebo. A subsequent analysis revealed beneficial results only for a small subset of patients (17% of the total) who had a HER2-positive disease. This subgroup presented better disease-free survival and lower mortality with metformin use vs. a placebo. The benefits seen in this HER2-positive subgroup was limited to patients with any C allele of the rs11212617 single-nucleotide variant [42].

Clinical trials regarding metformin use in prostate cancer were initiated enthusiastically, following encouraging results of in vitro studies. Although some retrospective studies presented a better oncological result in diabetic prostate cancer patients taking metformin [4,43], again, these results were not confirmed by metformin treatment in nondiabetic prostate cancer patients. Metformin addition (850 mg twice a day) to docetaxel for metastatic castration-resistant prostate cancer did not improve outcomes in nondiabetic patients, as per the French TAXOMET trial [44]. Another possible research direction for metformin comes from adverse reactions to androgen-deprivation therapy, which is frequently employed in prostate cancer management, such as obesity, hyperinsulinemia, insulin resistance, and type 2 diabetes. These metabolic disturbances are known as important factors for biochemical failure after prostatectomy and radiotherapy; thus, metformin’s benefits for weight gain and insulin resistance are worth mentioning [45]. Currently, there is great interest in the results from a standard of care plus metformin arm of the large STAMPEDE trial, which is still recruiting to investigate this combination in castration-sensitive prostate cancer.

Shenouda et al. confirmed the safety and tolerability of metformin use (850 mg twice a day for 14 days prior to radiotherapy and then over 6 weeks of radiotherapy) in glioblastoma patients and validated previous results regarding favorable outcomes, particularly those with low methylation levels of MGMT [46]. A pooled analysis of data from 1731 individuals with glioblastoma from the randomized AVAglio, CENTRIC, and CORE trials revealed that metformin use for diabetic-associated pathologies did not correlate with any improvement in OS or PFS [47].

## 3. Mebendazole

The most common presentation of mebendazole is in 100 mg tablets. As an anthelminthic with low-dose administration, side effects usually consist of abdominal pain and discomfort, flatulence, and diarrhea. Neutropenia and abnormal liver function tests have rarely been reported in high-dose regimens [48].

The main antitumor mechanisms of mebendazole:

- Behaves like colchicine and chemotherapy drugs, such as taxanes and vinca alkaloids, inhibits tubulin polymerization, and causes mitotic arrest, selectively in tumor cells without major side effects [49];
- Significantly reduces tumor angiogenesis, with no effect on microvessel density in normal tissue [8];
- Inhibits the hedgehog pathway and Gli1, BRAF, and MEK expression, with a reduction in tumor proliferation. Higher concentrations of mebendazole determine apoptosis through phosphorylation of Bcl-2 and activation of the mitochondrial pathways. P21 and p53 pro-apoptotic proteins are elevated after mebendazole treatment;
- Inhibits kinases, such as TNIK and VEGFR, thereby increasing tumor infiltration with CD8 T cells for better tumor control;
- Stimulates pro-inflammatory response and a pyroptosis type of cell death through activation of the NF-κB/NLRP3/GSDMD pathway;
- Activates an antitumor immune response through increased pro-inflammatory M1-phenotype cytokines (i.e., IL-1β, IL-8, IL-6, and tumor necrosis factor) and T-cell-attracting chemokines [48].

In vitro use of mebendazole and two other class representatives, flubendazole and fenbendazole, inhibit proliferation of glioblastoma cells in a dose-dependent manner by suppressing DNA synthesis, modulating the expression of key epithelial–mesenchymal transition markers, and inducing cell cycle arrest at the G2/M phase via the pro-apoptotic genes in the P53/P21/cyclin B1 pathway. Furthermore, the pyroptosis type of cell death, also known as cellular inflammatory necrosis, was found in the above-treated glioblastoma cells, through the NF-κB/NLRP3/GSDMD pathway. Tumor growth inhibition was confirmed, in vivo, in a nude mouse U87 cell xenograft model [50]. In a preclinical study from 2018, mebendazole was tested on rat models of glioblastoma and medulloblastoma. Even if mebendazole administered in monotherapy caused cytotoxicity, when combined with radiotherapy, colony formation was reduced even further and cleaved caspase-3 levels were higher. Both mebendazole monotherapy and the association had a survival advantage in the in vivo investigation, with an increase in apoptosis and reductions in tumor cell and vascular growth [51].

The association between temozolomide and other drugs could be an option for those patients who have resistant tumors to radiotherapy or chemotherapy (temozolomide), respectively, for those with low levels of FGFR3 and AKT2. Kipper et al. demonstrated that triple therapy with mebendazole plus topotecan plus bevacizumab versus temozolomide was superior at decreasing cell numbers in different cultures. As a result of these findings, the triple combination of temozolomide, vinblastine, and mebendazole may be a viable treatment option for gliomas with low expression of FGFR3/AKT2 [52].

Mebendazole demonstrated a decrease in breast-cancer-initiating cell levels, as it promoted double-stranded breaks, apoptosis, and arrested cells in the G2/M phase of the cell cycle [10]. Similarly, in triple-negative breast cancer cells and radiotherapy-resistant breast cancer cells, mebendazole determined DNA damage, downregulation of CD44 stem cell marker, and downregulation of OCT3/4 and ESM-1 tumor progression markers. Triple-negative breast cancer patients face short survival due to the tumor developing treatment resistance. The authors concluded that the addition of mebendazole to radiotherapy may provide a synergistic antitumor effect as part of a treatment strategy for triple-negative breast cancer [53].

Among the cancer types that mebendazole was proven against were human papillary and anaplastic thyroid carcinoma cells in vitro. By activating the caspase-3 pathway, mebendazole enhanced the percentage of cells in G2/M cell cycle arrest, triggered late-stage apoptosis in papillary (B-CPAP) and anaplastic (8505c) cell lines, decreased migratory and invasive capacity in aggressive 8505c cells. Another benefit of daily oral mebendazole was that it prevented existing thyroid cancers from spreading to the lungs and all these coupled with a known safety profile of high-dose oral mebendazole [49].

In association with anti-inflammatories, mebendazole significantly reduced tumor initiation and development in colon cancer models [54], and a similar effect was hypothesized in pancreatic cancer mice models. Both the KrasLSL.G12D/+ and the Pdx1-Cre (KC) mouse models of cerulean-induced inflammatory pancreatitis and the KrasLSL.G12D/+, Tp53R172H/+, and Pdx1-Cre (KPC) animal types of advanced pancreatic cancer responded to mebendazole administration by reducing pancreas weight, dysplasia, and intraepithelial neoplasia development. Mebendazole displayed a local, antitumor effect but also acted against liver metastases [11].

In addition to occasional isolated reports of mebendazole’s clinical use as a desperate attempt for terminally ill patients, most of the results stem from in vitro studies, with a few phase 1–2 clinical prospective studies reporting or in progress. One such phase 2 study, initiated by Mansoori et al., investigated the safety and efficacy of individually dosed mebendazole in patients with advanced gastrointestinal cancer. Although well tolerated, the study stopped early due to the inability to maintain serum concentrations, even at 4 g of mebendazole per day, and a lack of clinical response at best, considering all patients presented rapid disease progression [55]. Another clinical study, expanding on the in vitro benefits of mebendazole in gliomas, explored the association between mebendazole and temozolomide (200 mg/m^2^ once daily on days 1–5 of a 28-day cycle with mebendazole 1600 mg thrice daily) or lomustine (110 mg/m^2^ every 6 weeks with mebendazole 800 mg thrice daily) for recurrent glioblastomas. With almost 30% of the enrolled patients having a poor performance status, the study failed to achieve the 9-month OS objective [56].

## 4. Atorvastatin

Tumor cell membranes contain essential phospholipids made from fatty acids, similar to healthy cells. These lipids are obtained by the endogenous metabolites through the mevalonate pathway, making the tumor dependent on this process. Furthermore, multiple tumors demonstrate increased fat uptake, storage, and production, which lead to tumor progression.

The main atorvastatin antitumor mechanisms:

- Inhibition of cholesterol and the accompanying metabolite, 27-hydroxycholesterol, through the mevalonate pathway. The latter behaves as an estrogen receptor, important in estrogen-driven tumors;
- Inhibition of Akt/mTOR and activation of the MAPK pathway [12];
- Reduction of isoprenoid production, intermediate metabolites in the cholesterol pathway, which leads to a decrease in the cell-growth-stimulating proteins Ras, Rac, and Rho. Depletion of isoprenoid driven growth proteins induces apoptosis;
- Suppression of tumor development associated inflammation through inhibition of pro-inflammatory cytokines;
- Stimulation of autophagy and ferroptosis, associated with inhibition of the mevalonate pathway, as a type of programmed cell death, characterized by iron overload and lipid reactive oxygen species accumulation [13].

Thus, in the case of cancer patients with high amounts of lipids, the proteins involved in this process may be excellent chemotherapeutic targets [57]. Inhibiting this vital process could be beneficial in cancer cells, as they are usually rapidly proliferating without affecting too much the slower reproducing healthy cells. Inhibition of cholesterol synthesis by statins impacts cell membrane integrity, protein synthesis, cell signaling, and cell cycle progression [58].

For patients with head and neck cancer and hyperlipidemia, atorvastatin use (dose not available) improved OS and CSS compared to hyperlipidemia patients not taking atorvastatin, but this first category also recorded benefits from atorvastatin use when compared to patients without hyperlipidemia. Therefore, it is possible that statins disrupt cancer cell metabolism and proliferation without increasing cancer-related toxicity events [59]. These are radically different results from those obtained with another electron chain inhibitor, the above-described metformin, which showed far poorer results in combination with radiotherapy for lung cancer patients [40,41]. From this point of view, it would be of great interest to test atorvastatin’s antitumor effects in patients without hyperlipidemia.

There are limited studies on the effect of cotreatment with atorvastatin and radiotherapy. In a study by Hosseinimehr et al., the radiosensitizing effects of atorvastatin were evaluated by flow cytometry and antiproliferation assays for breast and lung cancer cells. The apoptosis rate of MDA-MB-231 breast cancer cells and A-549 lung cancer cells was increased in the atorvastatin group as well as in the irradiated group with a higher benefic effect recorded from a combination of both treatments. Atorvastatin increased reactive oxygen species production, thus further augmenting apoptosis in irradiated cells. An atorvastatin dose concentration of 10 µM diluted in culture medium proved a radiosensitizing effect on the above-tested tumor types and a radioprotective effect on previously irradiated normal lymphocytes [16,60].

For melanoma cell lines, mitochondrial OxPhos inhibitor IACS-010759 (IACS) was tested in a combination strategy with atorvastatin. Acting separately, both compounds induced a small increase in G2/M and sub-G1 phase cells, which further increased following IACS + atorvastatin treatment. Treatment combination downregulated pro-growth signaling proteins, such as phospho-Rb_Ser807_811, upregulated growth inhibitory/cell death proteins, and inhibited the IACS-induced AKT activation, resulting in the downregulation of the cell cycle and activation of cell death proteins. In the high-fat keto diet-fed mice, atorvastatin treatment induced minimal inhibition of tumor growth in BRAF inhibitor-acquired-resistant (A375R1) and intrinsic BRAF inhibitor-resistant (UCSD354L) melanoma cell lines compared with the vehicle, as was also observed in mice fed with a regular diet. The IACS treatment, however, induced potent tumor regressions within 20 days and IACS-010759 + atorvastatin completely eradicated both tumor types [61].

Atorvastatin administration also showed a benefit regarding prostate cancer cells. In vitro experiments with a plate colony formation assay and a multitarget/single-hit model confirmed an enhanced inhibiting effect of ionizing radiation on prostate cancer cells previously treated with atorvastatin. The same effect was reproduced on a prostate cancer cell-induced xenograft tumor in mice. The authors suggested that atorvastatin modulated the tumor cell colony formation through regulation of Bcl-2 and MSH2 molecule expression [62].

Among all oncological treatment options, besides the above associations with radiotherapy and targeted treatments, atorvastatin was also tested in combination with zoledronic acid, an agent usually involved in the treatment of bone metastases due to the osteoclasts’ inhibiting properties. Recently, in research, zoledronic acid proved the mevalonate pathway’s inhibiting properties, similar to atorvastatin. The effect of this combination was assessed on the adhesion of two ER-negative breast cancer cell lines to extracellular matrix proteins, which is an intermediary step in the metastatic process. The authors concluded that atorvastatin suppressed the adhesion of breast cancer cells, with a higher potency than that of bisphosphonates. The clinical implications could be significant as the high concentrations of zoledronic acid required to achieve similar results may limits its potential [14].

Preclinical studies indicated that statins inhibited glioma cell proliferation, migration, and invasion in a concentration-dependent manner. Synergistic antiglioma effects were observed when statins were combined with other anticancer therapies. Clinical observational studies showed an inverse, non-statistically significant, association between statin use and the incidence rate of glioma [63]. For patients with glioblastoma, a clinical study involving atorvastatin treatment association (40 mg daily for 3 weeks, followed by 80 mg until disease progression or unacceptable toxicity) did not improve 6 months progression-free survival, although this study identified high low-density lipoprotein levels as an independent predictor of poor cancer-related outcomes [64].

The SPECTRE phase II trial combined atorvastatin (40 mg daily for 6 weeks) and androgen-deprivation therapy in castration-resistant prostate cancer patients. Half of the patients (6 out of 12 patients) presented a decrease in their PSA velocity with minimal side effects. The data are suggestive of disease stabilization following a short-course statin treatment [65]. The ongoing ESTO2 phase 3 randomized double-blind trial plans to enroll 400 patients with metastatic or recurrent prostate cancer to receive 80 mg daily atorvastatin vs. placebo during androgen-deprivation therapy. The objective is to explore whether atorvastatin delays development of castration resistance.

Other studies aimed to investigate the radioprotective effect of atorvastatin or mitigate the acute adverse effects during radiotherapy. On this topic, one study looked into patients undergoing radiotherapy for breast cancer. Frequently met side effects, such as itching, breast edema, and pain, were significantly reduced in patients who used atorvastatin ointment during radiotherapy [66]. Furthermore, in addition to the aforementioned in vitro effect against breast cancer cells, atorvastatin (median dose: 20–40 mg) may play a role in mitigating anthracyclines/trastuzumab-induced cardiotoxicity often seen in breast cancer patients [67]. Others demonstrated that atorvastatin reduced the side effects of radiotherapy on lymphocytes, testis, and kidney. Anti-inflammatory-enhanced endogenous antioxidant, inhibition of lipid peroxidation, and caspase-3 are the main mechanisms involved in the radioprotective effect of atorvastatin on normal cells and tissues [16,17,18].

## 5. Propranolol

Modulators of beta-adrenergic signaling are often involved in the treatment of cardiovascular pathology and esophageal varices.

The rationale behind propranolol’s proposed antitumor effect:

- Beta-agonists stimulate cell proliferation in lung cancer cells by increasing IL-6 expression and inhibiting tumor suppressor LKB1 in EGFR-positive lung adenocarcinoma tumors [68]. Chronic adrenergic stimulation impairs the response to chemotherapy. Catecholamines inhibit chemotherapy-induced apoptosis in ovarian cancer cells through DUSP1 overexpression [69];
- Various tumor types, such as angiosarcoma, liposarcoma, and leiomyosarcoma, as well as other vascular lesions express beta-AR. The discovery of beta-blockers’ efficacy in hemangioma treatment led to their increased use in drug-refractory cancers [70];
- Propranolol reduces tumor angiogenesis and has immunostimulatory effects.

Observational studies that investigated the impact of beta-blocker use in breast, pancreas, and colorectal cancer present conflicting results. The largest meta-analysis concerning beta-blocker use in ovarian cancer concluded that post-diagnostic beta-blocker administration was not associated with the prognosis of patients with ovarian cancer [71].

A prospective nonrandomized study of propranolol (80 mg daily) in the adjuvant setting of resected melanoma found an 80% decrease in melanoma relapse [72], but also in metastatic melanoma, the use of specific therapy and nonselective beta-blockers led to better overall survival compared to selective beta-blockers [73]. Beta-blockers proved useful in association with certain chemotherapy drugs by enhancing their antiproliferative and antiangiogenic effects in tumor-bearing animals [74,75]. In different soft tissue sarcomas, propranolol, doxorubicin, and docetaxel association resulted in an increase in therapy response of which angiosarcoma and liposarcoma were the most sensitive. One possible explanation is the increased expression of P-glycoprotein after concomitant propranolol and doxorubicin administration and subsequent rise in doxorubicin intracellular levels. One clinical case with metastatic soft tissue sarcoma was reported with a favorable outcome in the form of stable, prolonged disease after docetaxel and propranolol treatment (40 mg daily for 18 months) [70]. SVR mouse angiosarcoma cells displayed signs of apoptotic response after propranolol treatment, such as increased p53, cleaved caspase 3, and cleaved PARP. Cell co-culture experiments with SVR angiosarcoma cells grown together with stained human dermal microvascular endothelial cells revealed the selective cytotoxic effect for tumor cells [76]. Nonselective beta-blockers are superior to selective beta-blockers, regarding angiosarcoma cancer inhibition, as well as for other tumor sites such as breast, ovarian, and liver [77,78,79]. This leads to increased outcomes (disease-free survival of 9 months vs. known data of 3–6 months, overall survival of 36 months vs. previous reports of 12 months) using propranolol (20–100 mg daily) in metastatic angiosarcoma patients [80]. In addition, combined with COX-2 inhibitors, propranolol (20 mg twice daily five days before surgery; 80 mg twice daily on the day of surgery; 20 mg twice daily for five post-operative days) displayed inhibitory effects related to metastases and disease relapse in a clinical study regarding breast cancer patients [81].

Pasquier et al. revealed that propranolol could be effectively associated with vinblastine-based metronomic chemotherapy in patients diagnosed with advanced, metastatic or relapsed angiosarcoma. Despite the small number of enrolled patients, complete and partial response cases were recorded. Their in vitro experiments pointed towards dose-dependent antiproliferative effects of propranolol (40 mg twice daily) against transformed endothelial cells. Alternative antitumor mechanisms have been suggested when using propranolol alone such as angiogenesis inhibition and immunostimulatory effects [82]. For tumors exhibiting beta-adrenergic receptors ARB1, ARB2 and ARB3, propranolol alone reduced the proliferation index by 34% [83]. Breast cancer patients were monitored after resection in a randomized triple-blind clinical trial in which markers of metastasis were inversely associated with the administration of propranolol (80–160 mg escalating dose), 7 days before surgery [84].

The association between an anthracycline chemotherapy drug, doxorubicin, frequently used in breast cancer treatment, and propranolol reduced the percentage of the cell population residing in the G2/M phase of the cell cycle and increased the sub-G1 cell population, representing dead or dying cells [85]. Circulating markers for predicting response to anthracycline chemotherapy in preoperative setting are already known [86] and related to this it would be interesting to investigate the patient sub-categories that would benefit most from the combination of anthracycline and beta-blockers. Cancer-specific mortality was found to be lower after the use of beta-blockers in observational studies of patients with prostate and pancreatic cancer but with marginal to no benefit in ovarian cancer [19].

**Table 1:** Main in vitro, in vivo, and clinical results in oncology for metformin, mebendazole, atorvastatin, and propranolol. Clinical prospective studies are in bold.

Tumor Site	Metformin	Mebendazole	Atorvastatin	Propranolol
**Breast**	Metformin and doxorubicin erased mice tumors and prevented recurrence [32]; **no clinical benefit, except for a small category of HER2+ patients; increased toxicity** [42]	Cell cycle arrest in the radiosensitive G2/M phase in triple-negative cancer cells [10]	Apoptosis and autophagy were both involved in tumor cell death [87]; impaired adhesion of breast cancer cells after atorvastatin and zoledronic acid exposure [14]; enhanced tumor radiosensitivity, exhibited radioprotective effect on normal cells [60]; **atorvastatin ointment reduced radiotherapy toxicity** [66] statins decreased the risk of cardiotoxicity after anthracycline/trastuzumab exposure [67]	**Markers of metastasis were inversely associated with the administration of propranolol before surgery [84];** **Combined with COX-2 inhibitors, propranolol inhibits metastases, and disease relapse** [81]
**Prostate**	Tumor growth delay in mice [27]; improved oxygenation in the prostatic tumor [4]; better oncological results in diabetic prostate cancer patients taking metformin [43] **metformin and docetaxel for metastatic prostate cancer did not improve outcomes in nondiabetic patients** [44]	Synergistic effect with docetaxel increasing apoptosis and reducing tumor growth [88]	ATV inhibited HIF-1α protein expression for increased radiosensitivity of prostate cancer cells [89]; atorvastatin and caffeine induced apoptotic death by downregulating phospho-Akt, phospho-Erk1/2,and anti-apoptotic Bcl-2 [90]; activation of LC3 transcription by atorvastatin induced autophagy in prostate cancer cells [91] **Decreased PSA velocities after statin treatment in castration-resistant prostatic cancer patients** [65]	Cancer-specific mortality was found to be lower after the use of beta-blockers in observational studies [19]
**Leukemia**	Sensitized leukemic cells to cytotoxic lymphocytes; delayed growth of a fast-growing lymphoma in vivo in the presence of an anti-CD20 mAb [92]	Gli inhibitor with strong antileukemic activity [93]; Inhibited chemoresistant T-ALL cells both in vitro and in vivo [94]	Mevalonate-YAP axis inhibition by atorvastatin suppresses K562 and HL60 cells [95]	
**Ovarian**		Inhibited growth of ovarian cancer cell cultures [96]	Ovarian cancer cells apoptosis was induced by statins through JNK activation and stimulation of Bim expression [97]	Longer median OS was observed among users of a nonselective beta-blockers compared with nonusers [78]; marginal benefit in observational studies [19]; post-diagnostic exposure to β-blockers was associated with a significant decrease in OS [98]
**Liver**	No survival benefit in diabetic patients undergoing metformin treatment [99]	Augmented sensitivity to sorafenib [100]	Atorvastatin inhibited the PI3K/Akt pathway and stimulated tumor cell death [101]	Nonselective beta-blockers may reduce risk of hepatocellular carcinoma [77]
**Colorectal/gastrointestinal**	Inhibited the growth of intestinal polyps [34]	Mebendazole and anti-inflammatories reduced tumor initiation [54]; **All patients presented disease progression** [55]	Post-diagnostic statins did not associate with improved PFS or OS [102]; statin use was a favorable prognostic factor [103]; no decrease in rectal cancer risk [104]	Activated autologous CD8+ T cells and decreased the expression of p-AKT/p-ERK/p-MEK in mouse tumor models [105]; no association between post-diagnostic β-blocker and specific mortality [106]
**Angiosarcoma/soft tissue sarcomas**	Increased survival in both nondiabetic and diabetic patients with soft-tissue sarcoma [107]	Blocked anticancer activity of the disulfiram and metformin combination, likely by NF-κB stimulation [108]		Displayed signs of apoptotic response after propranolol treatment [76]; propranolol reduced proliferation by 34% [83]
**Melanoma**	VEGF inhibitors and metformin synergized to suppress BRAF-mutant tumors [109]; Blocked exosome-mediated miR-34a secretion and sensitized these cells to cisplatin [110]	Bcl-2 inactivation led to apoptosis in chemoresistant melanoma cells [111]; Mebendazole and trametinib suppressed refractory melanoma [6]	Mitochondrial OxPhos inhibitor with atorvastatin reduced cell growth in BRAFi-resistant melanomas [61]	**An 80% decrease in melanoma relapse** [72]; better overall survival when compared to selective beta-blockers [73]
**Lung**	**Worse local control and increased side effects in the metformin arm [40];** **Metformin was well tolerated but no oncological benefit** [41]		Enhanced tumor radiosensitivity, exhibited radioprotective effect on normal cells [60]; better tumor response and longer PFS in patients with NSCLC treated with immune checkpoint inhibitors [112]	No association between betablocker use and improved overall survival in lung cancer [113,114]; increased PFS in patients treated with immune checkpoint inhibitors and beta-blockers [115]
**Glioblastoma**	In vitro decreased proliferation; autophagy and apoptosis; inhibition of the mTOR pathway [39]; **no oncologic benefit in diabetic patients** [47]	Vinblastine and mebendazole potentiate temozolomide in resistant gliomas [52]; **addition of mebendazole to temozolomide or lomustine failed to achieve the 9 month OS objective** [56]	**Treatment association was not shown to improve 6 month progression-free survival** [64]	Suppressed glioblastoma cell proliferation and induced Notch1 expression [116]
**Thyroid**	Metformin antitumor activity depended on the glucose level [38]; decreased thyroid cancer risk in diabetic patients [117]	Enhanced the percentage of cells in G2/M cell cycle arrest; prevented lung metastases from thyroid cancers [49]	Statins inhibited the growth of thyroid carcinoma cells in vitro [118]; Increased thyroid cancer risk in female patients [119]	Induced apoptosis of 8505C thyroid cancer cells in vitro and in vivo [120]
**Pancreas**	Better survival for patients after pancreatectomy [121]; inconsistent results in various studies [122]	Inhibited pancreatic tumor development [11]	Inhibited proliferation, migration, and invasion; induced G1-phase apoptosis in tumor cells [123]; associated to reduced pancreatic adenocarcinoma risk [124]	Cancer specific mortality was found to be lower after the use of beta-blockers in observational studies [19]
**Head and neck**	Inhibited proliferation but decreased cisplatin’s antitumor effect; HPV-negative had higher apoptosis than HPV-positive [125]	More potent antiproliferation activity in cancer cells than that of cisplatin [126]	**Improved OS and CSS in hyperlipidemic patients with primary disease in the oral cavity and oropharynx** [59]	No evidence that β-blockers can decrease the risk of head and neck cancer [127]

## 6. Conclusions

All of the above pharmacological agents present key mechanisms of action found to interfere with cancer cell metabolism. Most of the published studies referring to the four drugs were underpowered and lacked conclusive evidence for the use of the study’s drugs alone without the confounding impact of concomitant chemotherapy.

It noteworthy that, especially for metformin and mebendazole, most of the recorded antitumor effects were dose-dependent, employing much higher doses than the ones typically used in their already approved clinical settings. For example, the dose for mebendazole for anthelminthic purposes ranges from 100 mg single dose to 100–200 mg per day, whereas in the off-label treatment of leukemia or high-grade glioma, study designs employed doses between 50 and 200 mg/kg/day. This is particularly important for most of the retrospective studies, where patients took our drugs of interest for their standard clinical indications and not for cancer treatment.

One shortcoming of most of the above clinical studies is that the use of repurposed drugs in oncology is usually a desperate attempt, initiated only after failure of all the approved lines of treatment.

Despite the wealth of results from observational and preclinical studies, the only solid data from clinical, prospective studies confirm metformin-related oncological benefits for only a small subset of patients with HER2-positive breast cancer and early-stage colorectal cancer; metformin-related detrimental/lack of effect for lung, breast, prostate cancer, and glioblastoma. Atorvastatin’s oncological benefits were found in patients with head and neck cancer, with a trend towards radioprotection of critical structures, thus supporting the role of atorvastatin as a promising agent for concomitant association with radiotherapy. Propranolol-related increased outcomes were seen in patients with melanoma, breast cancer, and sarcoma.

Many clinical or observational studies failed to properly monitor patients’ comorbid conditions or other concomitant medications. It is well known that these four drugs of interest are usually administered for benign conditions frequently met at the same time, such as the use of beta-blockers for cardiovascular pathology together with statins and metformin for metabolic syndrome, thus providing anticancer pathways more intricate than most studies manage to account for.

## Data Availability

Not applicable.

## Abbreviation

VEGFR	vascular endothelial growth factor receptor
VEGF	vascular endothelial growth factor
BRAF	v-raf murine sarcoma viral oncogene homolog B1
ETC	electron transport chain
OS	overall survival
PFS	progression-free survival
LKB1	liver kinase B1
DNA-PKcs	DNA-dependent protein kinase, catalytic subunit
PI3K	phosphoinositide 3-kinases
Akt	serine-threonine protein kinase
MGMT	O6-alkylguanine DNA alkyltransferase
NLRP3	nucleotide-binding oligomerization domain-like receptor pyrin domain-containing 3
FGFR3	fibroblast growth factor receptor 3
ARB	adrenergic receptor
ESM-1	cancer progression-related protein level
PARP	poly (ADP-ribose) polymerase
B-CPAP	poorly differentiated thyroid gland carcinoma
Gli1	glioma-associated oncogene
Beta-AR	beta-adrenergic receptor
EGFR	endothelial growth factor receptor
Bcl2	B-cell lymphoma 2
TNIK	TRAF2- and NCK-interacting protein kinase	
AMPK	adenosine monophosphate-activated protein kinase	
HMG-CoA	3-hydroxy-3-methylglutaryl coenzyme A reductase	
FDA	US Food and Drug Administration	
HER2	human epidermal growth factor receptor 2	
IGF-I	insulin-like growth factor I	
TORC1	target of rapamycin complex 1	
CSCs	circulating tumor cells	
Redd1	regulated in development and DNA damage responses 1	
mTOR	mammalian target of rapamycin	
NF-κB	nuclear factor kappa B	
GSDMD	gasdermin D	
OCT3/4	octamer-binding transcription factor	
COX-2	cyclooxygenase-2	
IL-6	interleukin 6	
CSS	cancer-specific survival	
Stat3	signal transducer and activator of transcription 3	
ER	estrogen receptor	
DUSP1	dual specificity phosphatase 1	
OxPhos	oxidative phosphorylation	
MSH2	MutS Homolog 2 protein-coding gene	
